# Cholestatic hepatitis as a rare manifestation of *Mycoplasma pneumonia* in an adult: A case report

**DOI:** 10.1097/MD.0000000000044766

**Published:** 2025-09-19

**Authors:** Muhammad Husnain Ahmad, Ali Gohar, Haseeb Mehmood Qadri, Bilal Ahmed, Masab Ali, Ayesha Ayman, Muhammad Waqar Khaliq, Syed Saqlain Haider Shah

**Affiliations:** a Department of Internal Medicine, S Tentishev Asian Medical Institute, Kant, Chuy Province, Kyrgyzstan; b Department of Internal Medicine, Lahore General Hospital, Lahore, Punjab, Pakistan; c Department of Neuroscienes, Pakistan Institute of Neurosciences, Lahore, Pakistan; d Department of Internal Medicine, Punjab Medical College, Faisalabad, Punjab, Pakistan; e Department of Transplant Surgery, Pakistan Kidney and Liver Institute and RC, Lahore, Punjab, Pakistan; f Department of Internal Medicine, Al Raazi Hospital, Lahore, Pakistan.

**Keywords:** atypical pathogen, cholestatic hepatitis, community-acquired infection, *Mycoplasma pneumonia*, respiratory illness

## Abstract

**Rationale:**

*Mycoplasma pneumonia*, a common cause of community-acquired respiratory infections, is rarely linked to extrapulmonary complications like cholestatic hepatitis.

**Patient concerns:**

We report a case of a 48-year-old male who developed jaundice after a respiratory illness. Initial workup revealed cholestatic hepatitis with elevated bilirubin and liver enzymes, while common causes of hepatitis were ruled out.

**Diagnoses:**

Elevated pro-B type natriuretic peptide levels initially raised suspicion for heart failure, but cardiac evaluations were unremarkable. Chest X-ray showed bilateral infiltrates and pleural effusion, suggesting an underlying respiratory infection. Given the patient’s respiratory symptoms, an atypical pathogen was suspected. Polymerase chain reaction for *M. pneumonia* DNA confirmed the diagnosis.

**Interventions:**

The patient was treated with azithromycin, resulting in significant improvement.

**Outcomes:**

One-month follow-up showed normalized liver function and resolution of symptoms.

**Lessons:**

This case demonstrates that hepatobiliary involvement of *M. pneumonia* can occur in the immunocompetent adult. By excluding the other causes of cholestatic hepatitis and comparing the results of impaired liver function test with clinical and radiological findings characteristic of *M. pneumonia* infection, hepatobiliary involvement of *M. pneumonia* infection was confirmed. Only a small number of adult instances of *M. pneumonia*-associated cholestatic hepatitis have been documented. This case adds to the scant literature on *Mycoplasma*-associated cholestatic hepatitis and highlights the necessity for physicians to rule out *M. pneumonia* as a differential diagnosis in patients with respiratory illness and hepatic dysfunction.

## 1. Introduction

*Mycoplasma pneumonia* is a small, cell wall-lacking bacterium transmitted via respiratory droplets.^[1]^ It causes year-round infections, peaking in summer and early fall, primarily affecting children aged 5 to 15.^[2]^ While pulmonary involvement is often mild, 3% to 10% of patients develop *pneumonia*, while quarter of patients may experience extrapulmonary complications.^[3]^ One extremely rare extrapulmonary manifestation is cholestatic hepatitis that leads to jaundice after *Mycoplasma* infection,^[1]^ likely due to immune-mediated damage or autoimmunity.^[4]^ Diagnosing *M pneumonia* involves polymerase chain reaction (PCR) or specific immunoglobulin M (IgM) detection, as culture takes 1 to 2 weeks yet remained gold standard.^[5]^ Radiographic findings vary, often showing pleural effusions and diffuse infiltrates.^[2]^ Treatment includes macrolides with piperacillin-tazobactam, resolving both respiratory and hepatic symptoms.^[4]^ We presented a case of middle aged patient with cholestatic jaundice, emphasizing the importance of recognizing *M pneumonia* as a rare cause of cholestatic hepatitis and differentiating it from other causes of acute hepatitis.

## 2. Case description

A 48-year-old male without any comorbidities presented to the medical emergency department of tertiary care hospital with a 2-week history of fever, followed by 1 week of cough, shortness of breath, and yellow discoloration of the skin and sclera for 5 days. There was no history of alcohol or drug intake. His vitals at the time of presentation were blood pressure: 120/70 mm Hg, heart rate: 110 bpm, temperature: 100°F, respiratory rate: 29/min, oxygen saturation: 50% on room air. On examination, the patient was conscious, oriented, and jaundiced. Chest auscultation revealed bilateral harsh vesicular breathing and decreased air entry in the right lower zone. Abdominal examination showed tenderness in the right hypochondrium. Congestive cardiac failure was the provisional diagnosis. Emergency management included diuretics, urinary catheterization, and strict input/output monitoring.

**Figure 1.**
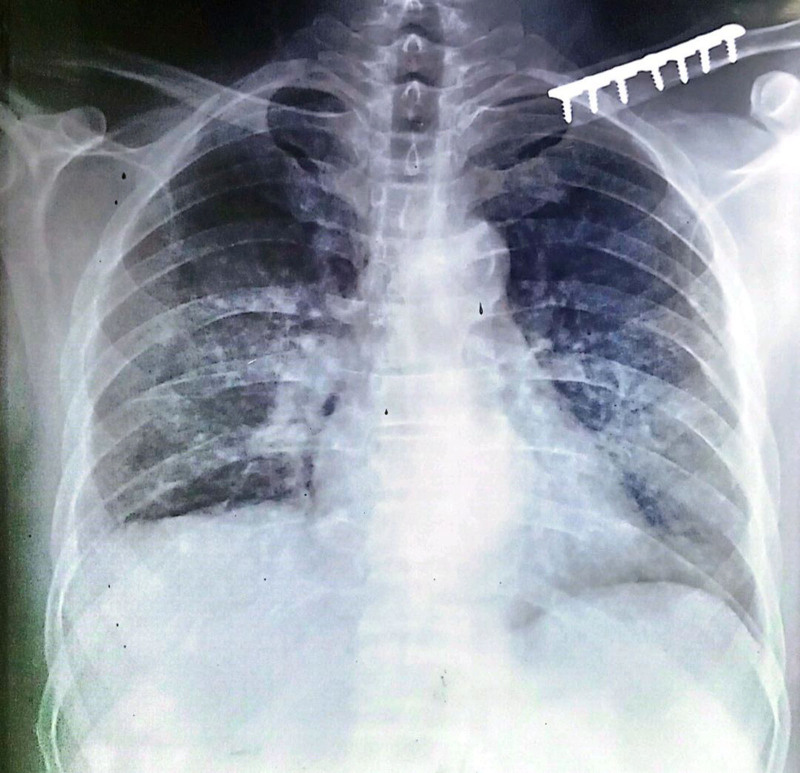
Chest x-ray (PA view) shows bilateral heterogeneous infiltrates and right sided pleural effusion. x-ray = radiograph.

The patient’s initial lab results indicated cholestatic hepatitis, with elevated total bilirubin (7.7 mg/dL), lactate dehydrogenase (1868 U/L), alanine transaminase (66 U/L), and alkaline phosphatase (354 U/L). Hemoglobin and leukocyte counts were within normal ranges, 12.9 g/dL and 10.8 × 10³/µL, respectively. The viral markers for hepatitis and serology for human immunodeficiency virus were negative. To rule out the possibility of acute viral hepatitis, repeat serological testing for hepatitis A, E, B, and C was performed after a 2-week interval. All results remained negative, further excluding viral hepatitis as a contributing factor. The autoimmune profile was unremarkable (Table 1). Cardiac evaluation that involved myocardial enzyme testing (CPK, CPK-MB or troponin) (Table 3) electrocardiogram (ECG) were normal ruling out myocarditis. Moreover, there was no appreciable left ventricular dysfunction on echocardiography (Table 2). Additionally, N-terminal pro-B type natriuretic peptide (Pro-BNP) levels were significantly elevated at 12,073 pg/mL, indicative of cardiac strain. However, absence of lower limb edema, increased jugular venous pressure and hepatomegaly clinically ruled out congestive heart failure. Chest x-ray (radiograph) showed bilateral heterogeneous infiltrates with right-sided pleural effusion (Fig. 1). Abdominal ultrasound was unremarkable.

**Table 1:** Autoimmune profile.

Test	Result
Hepatitis B surface antigen (HbsAg)	Negative
Anti-hepatitis C virus (Anti-HCV)	Negative
Anti-human immunodeficiency virus (Anti-HIV)	Negative
Antinuclear antibody (ANA)	Negative
Direct Coombs test	Negative
Indirect Coombs test	Negative
Hepatitis A virus IgM (HAV IgM)	Negative
Hepatitis E virus IgM (HEV IgM)	Negative
*Mycoplasma* IgM	Positive
B-type natriuretic peptide (Pro BNP)	12,073 pg/mL
PCR for *Mycoplasma* DNA	Positive
Cultures	No growth detected

ANA = antinuclear antibody, Anti-HCV = anti-hepatitis C virus, Anti-HIV = anti-human immunodeficiency virus, HAV = hepatitis A virus, HbsAg = hepatitis B surface antigen, HEV = hepatitis A virus, IgM = immunoglobulin M, PCR = polymerase chain reaction, Pro BNP = B-type natriuretic peptide.

**Table 2:** Echocardiogram.

Parameter	Reference range	Results
Aortic root diameter	20–37 mm	27 mm
Left atrium diameter	20–39 mm	34 mm
Left ventricular end diastolic diameter (LVEDD)	42–56 mm	44 mm
Left ventricular end systolic diameter (LVESD)	30–40 mm	28 mm
Interventricular septum (IVS) thickness	6–10 mm	12 mm
Left ventricular posterior wall (LVPW) thickness	6–10 mm	12 mm
Left ventricular ejection fraction (LVEF)	>55%	64%
Fractional shortening (FS)	30–45%	31%

FS = fractional shortening, IVS = interventricular septum, LVEF = left ventricular ejection fraction, LVEDD = left ventricular end diastolic diameter, LVESD = left ventricular end systolic diameter, LVPW = left ventricular posterior wall.

**Table 3:** Serial laboratory investigations.

Parameter	Reference range	At presentation	Day 5 of admission	1-mo follow-up
Total bilirubin (mg/dL)	0.2–1.2	7.7	3.4	1.0
ALT (U/L)	<41	66	52	35
ALP (U/L)	40–130	354	210	125
LDH (U/L)	140–280	1868	950	270
Pro-BNP (pg/mL)	<125	12,073	5000	110
Hemoglobin (g/dL)	13.0–17.0	12.9	12.8	13.4
WBC (×10³/µL)	4.0–11.0	10.8	8.7	7.5
CPK	10–120 mcg/L	55	43	45
CK-MB	0–3 ng/mL	1.2	1.1	1.2
Troponin I	<0.35 ng/mL	0.01	0.01	0.03

ALT = alanine transaminase, ALP = alkaline phosphatase, LDH = lactate dehydrogenase, Pro-BNP = N-terminal pro-B type natriuretic peptide, WBC = white blood cell.

**Figure 2.**
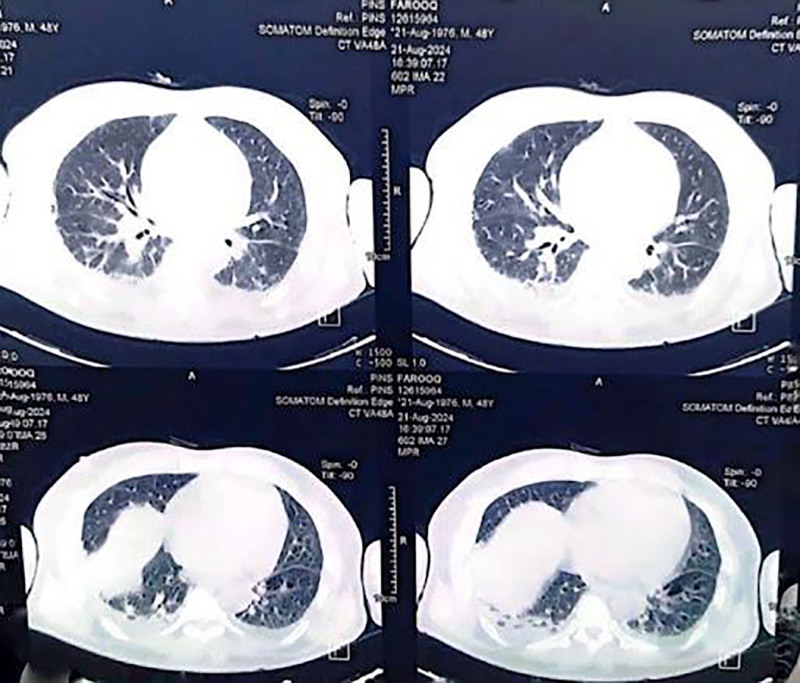
HRCT chest shows interstitial thickening and emphysematous changes in both lungs. HRCT = high-resolution computed tomography.

Based on the patient’s respiratory symptoms and chest x-ray findings, a pulmonology consultation was obtained, and serological testing for *M pneumonia* was performed. *Mycoplasma* IgM was positive, and a convalescent phase IgM titer performed after 3 weeks showed a fourfold rise, confirming the diagnosis of *Mycoplasma* infection. High-resolution CT of the chest showed interstitial thickening and emphysematous changes in both lungs (Fig. 2). To further establish the diagnosis, molecular testing via PCR for *M pneumonia* DNA was performed and yielded positive results. Bacterial and fungal blood cultures were negative, ruling out other infectious causes of hepatitis and myocarditis.

After confirmation of *Mycoplasma* on PCR, the patient was switched from piperacillin-tazobactam to 500 mg of intravenous azithromycin once daily for 2 days, followed by oral 500 mg once daily for next 8 days. A summary of serial laboratory investigations, including liver function tests, cardiac enzymes and Pro-BNP may be found in Table 3.

After starting the macrolide, the pulmonary infiltration resolved in 10 days. Additionally, liver function tests begin to improve. At the 1-month follow-up, the patient’s liver function tests had completely normalized, including serum bilirubin, alanine transaminase, alkaline phosphatase, and lactate dehydrogenase levels. Furthermore, the previously high Pro-BNP level dropped back into the normal reference range, supporting the resolution of both cardiac and hepatic stress.

## 3. Discussion

*M pneumonia* primarily causes mild respiratory infections,^[3]^ but can also impact nearly every organ, including the skin, nervous, hematologic, cardiovascular, and musculoskeletal systems.^[2]^ The exact mechanism behind hepatobiliary involvement in *M pneumonia* infection remains unclear. It is suggested that this involvement may arise either from direct infection of hepatocytes or bile duct epithelial cells, or as a consequence of immune-mediated processes. Additionally, hepatocellular damage might be linked to cross-reactive antibodies triggered by *Mycoplasma* infection, similar to its other extrapulmonary manifestations.^[6]^

Saraya et al noted that the highest infection risk is in individuals aged 5 to 20 years,^[5]^ but in our case patient is middle aged adult. This underscores the need for clinicians to consider *Mycoplasma* as a potential cause of cholestatic hepatitis in middle aged patients.

The diagnosis of *M pneumonia* infection is established through a combination of positive serological tests, including IgM and convalescent phase IgM titers, along with molecular confirmation via PCR. The link between *Mycoplasma* infection and the observed hepatobiliary dysfunction is further supported by ruling out other infectious agents such as hepatitis A, B, and C viruses, Epstein-Barr virus, and cytomegalovirus similar to the case reported by Grüllich et al^[6]^ Moreover, negative bacterial and fungal blood cultures helped exclude other potential infectious causes of hepatitis and myocarditis.

One strikingly distinctive aspect about our study included the elevation of pro-BNP however the echocardiography and ECG was normal hence excluding congestive cardiac failure. The elevation in Pro-BNP observed in this patient, despite normal echocardiographic and ECG findings, may reflect a systemic inflammatory response rather than intrinsic cardiac dysfunction. *M pneumonia* is known to induce cytokine-mediated endothelial injury, which can lead to transient myocardial strain and elevated natriuretic peptides without structural heart disease. This highlights the importance of interpreting elevated B-type natriuretic peptide levels in the context of the overall clinical picture.^[4]^ This elevation in pro-BNP and chest pain can also prompt physicians to consider heart failure in diagnostic picture highlighting the need to do all the required investigations for effective diagnosis and management.

In our case piperacillin-tazobactam along with azithromycin resulted in improvement in jaundice and stabilization of liver function tests while in case report of Kanagavelu et al similar regimen was used initially followed by ciprofloxacin and clarithromycin highlighting the fact that this combination of antibiotics regimen should be used by the physicians while encountering this rare pattern of cholestatic hepatitis case.^[1]^

A summary of previously reported adult cases of *M pneumonia*-associated cholestatic hepatitis is provided in (Table 4). Our case further contributes to this limited pool of literature by documenting complete resolution of liver function and normalization of Pro-BNP following targeted antimicrobial therapy.

**Table 4:** Summary of reported cases of cholestatic hepatitis in adults with *Mycoplasma pneumonia* infection.

Author (yr)	Age/sex	Presentation	Diagnosis	Treatment	Outcome
Present case (2024)	48/M	Fever, cough, SOB, jaundice	IgM and PCR positive for *M pneumonia*	Piperacillin-Tazobactam + Azithromycin	Liver function normalized
Kanagavelu et al^[1]^	35/M	Hemolysis, jaundice, pleuritic chest pain	IgM positive for *M pneumonia*	Piperacillin-Tazobactam, Ciprofloxacin	Improved after switch
Shin et al^[4]^	45/M	Hepatitis with respiratory symptoms	Serology and clinical features	Azithromycin	Complete resolution
Grüllich et al^[6]^	38/M	Fever, jaundice, fatigue, cough	IgM serology and PCR for *M pneumonia*	Macrolides (unspecified)	Full recovery
Khan and Yassin^[3]^	30/F	Jaundice, hemolysis, dyspnea	Serology	Supportive + Antibiotics	Full recovery

IgM = immunoglobulin M, PCR = polymerase chain reaction, SOB = shortness of breath.

## 4. Limitations

In our case, lack of standardized protocols and limited diagnostic facilities made it difficult to diagnose *M pneumonia*. First, it was misdiagnosed, and the right diagnosis was not made until further evaluation. Unfortunately, our hospital setting lacks advanced diagnostic equipment like PCR machines, which would have facilitated a more precise and quick diagnosis. The delay was also due to high cost of diagnostic tests and limited awareness among healthcare professionals. These limitations show that in order to properly manage *M pneumonia* in tertiary care hospitals, we need better diagnostic tools, standardized guidelines, and increased awareness.

## 5. Conclusion

This case provides insights into the diagnosis and management of cholestatic hepatitis secondary to *M pneumonia*. As a rare occurrence, it adds to the limited literature on this association. Early recognition and targeted treatment are essential for favorable outcomes, and further research is needed to deepen understanding and optimize management strategies.

## Acknowledgments

We would like to thank the team of clinicians who helped manage this case. We would like to thank the patient and his family members for their cooperation in bringing this case for the betterment of the scientific community.

## Author contributions

**Conceptualization:** Ali Gohar, Haseeb Mehmood Qadri, Ayesha Ayman.

**Data curation:** Ali Gohar, Haseeb Mehmood Qadri.

**Formal analysis:** Ali Gohar, Haseeb Mehmood Qadri, Masab Ali, Muhammad Waqar Khaliq.

**Funding acquisition:** Bilal Ahmed, Ayesha Ayman, Syed Saqlain Haider Shah.

**Investigation:** Haseeb Mehmood Qadri, Ayesha Ayman, Syed Saqlain Haider Shah.

**Project administration:** Bilal Ahmed.

**Resources:** Bilal Ahmed, Syed Saqlain Haider Shah.

**Software:** Bilal Ahmed, Syed Saqlain Haider Shah.

**Supervision:** Ali Gohar, Bilal Ahmed, Masab Ali, Muhammad Waqar Khaliq.

**Validation:** Ali Gohar, Muhammad Husnain Ahmad, Muhammad Waqar Khaliq.

**Visualization:** Muhammad Husnain Ahmad, Ayesha Ayman.

**Writing – original draft:** Ali Gohar, Haseeb Mehmood Qadri, Bilal Ahmed, Masab Ali, Ayesha Ayman.

**Writing – review & editing:** Masab Ali.
